# The Bacteriology of Diphtheria

**Published:** 1891-12-12

**Authors:** 


### The Practitioner's Mirror and Retrospect.

[*The Editor will be glad to receive offers of co-operation and contributions from members of the profession. All letters should be addressed to* The Editor, The Lodge, Porchester Square, London, W.]

### MEDICINE.

### THE BACTERIOLOGY OF DIPHTHERIA.

Unlike tuberculosis, the exact nature of the materies morbi in diphtheria has never been accepted as proved by the medical profession generally, but the elaborate investigations of the last year or two have almost conclusively proved that the Klebs-Löffler bacillus, is the essential cause of diphtheria, a fact of great practical importance. Löffler made a series of observations on twenty-five cases, in all of which he found the bacillus. Pure cultivations of them were made, and inoculations of them reproduced false membranes in various animals; and intravenous and subcutaneous injections of them gave the same results. But some doubt was thrown on the identity of the Löffler bacillus with the origin of true diphtheria, because (*a*) in no cases of his did post-diphtheric paralyses occur; (*b*) in some typical cases of diphtheria and bacillus was not found; (*c*) in a few cases the bacillus was found in health. Roux and Yersin next took up the study, and these observers, working in the Pasteur laboratory, with improved methods, found the true bacillus in every case of diphtheria examined, and were able to identify the non-pathogenic and pseudo-bacillus found in healthy mouths, and to distinguish it from the true bacillus. Diphtheritic paralysis also occurred in some of the inoculated animals, and when we remember that paralysis only occurs in about eleven per cent. of diphtheritic cases, its absence in Löffler's twenty-five cases is not so remarkable. More recently the aggregate results of examinations by Babes, D'Espine Otzmann, Paltauf, Beck, have shown that the Löffler bacillus was present in all, save one, of 122 cases examined, and absent in all non-diphtheritic cases. It may, therefore, be regarded as proved that the Klebs-Löffler bacillus is the infecting organism in diphtheria.

But other points of great practical importance have been demonstrated in regard to this bacillus. The bacillus never invaded the organs of the body; it exists only in the false membrane of the region inoculated, and is never found in the blood. This fact led to grave doubts as to the possibility of its being the real cause of the disease, but recently Roux and Yersin have found that all the phenomena of true diphtheria may be produced by the inoculation of cultures of the poison sterilised by filtration through porcelain filters, and it is now generally admitted that the symptoms of diphtheria are produced by the absorption into the circulation of the poisons secreted by the bacillus, and this poison Roux and Yersin have shown is the nature of a diastase; it is not a ptomaine and it is not of a crystalline character. Brieger and Fraenkel have shown that when inoculated into animals this poisonous substance is fatal in the dose of about one twenty-fifth of a grain for every two pounds of body weight. It will be remembered that, at a recent meeting of the Pathological Society, Dr. Andrew Wilson showed that in ordinary diphtheria a thin grey exudation preceded the membrane format on, and this exudation, he believed, contained the causative micro-organism. He inoculated tubes, and there first appeared a funnel-shaped depression in the gelatine, followed by liquefaction and the formation of an orange-coloured deposit. The germ proved on examination to be a pure cultivation of a small micrococcus. Pigeons were inoculated from this, and in them a true diphtheria was produced, with membrane exudation and paralysis. Acids retarded the growth of these micrococci. But, as Dr. William Hunter pointed out, these results entirely disagreed with the latest continental researches to which we have alluded. The practical points which have a direct bearing on the therapeutics of diphtheria, which are borne out by the pathological investigations to which we have so briefly referred, may be summed up: (1) Diphtheria is due to infection by a definite bacillus. (2) The bacillus grows and develops at the seat of inoculation only, and does not enter the tissues or circulation. (3) The symptoms of the disease are due to a toxalbumen secreted by the bacillus in the false membrane and absorbed into the circulation. This bears out the clinical observations that the extent of the fal e membrane is in proportion more or less to the severity of the symptoms. As regards the prophylaxis of diphtheria, Professor Löffler lately read a paper on the prevention of the spread of diphtheria, in which he summarised our present knowledge on this point. The bacillus of diphtheria is thrown out with the excretions of the affected mucous membranes and may be deposited on the patient's surroundings. Patients harbour micro-organisms able to produce an infection as long as a shred of membrane remains, and even some days after its complete disappearance. They should be strictly isolated while the excretions contain bacilli, and children kept from school for at least four weeks. The germs are visible in pieces of membrane in the dry state for for four or five months, and all objects about the patient, e.g., clothes, vessels, nurses' apparel, &c., ought therefore to be disinfected by boiling or steaming at 100 deg. C. The rooms are to be carefully cleaned, the floors washed with warm sublimate solution (1-1,000), and the walls and furniture rubbed with bread. Damp and badly-lighted houses appear favourable to the propagation of the virus. These ought to be put into proper condition, thoroughly dried, and have free entrance of light and air. The milk supply should be carefully supervised, as the bacilli grow well in this fluid at a temperature of 20 deg. C. Similar affections in the lower animals, as pigeons, fowls, calves, and swine, are not caused by the same germ as in man, and need not be regarded as sources of infection for human beings. Löffler considers that Dr. Klein has not yet proved the identity of a disease in cats with the diphtheria of man. Lesions of the mucous membrane of the primary tracts favour the entrance of the virus; but susceptible individuals may take it without that. It is important, therefore, during an epidemic to attend carefully to the cleansing of the mouth, throat, and nose of children, and mouth washes and gargles of aromatic water, or sublimate solution (1-10,000) are recommended.

He believes that a natural therapy of diphtheria can only be found by the study of the effect of different medicaments on the cultures of the diphtheria bacilli. Concerning the therapy, we must try to prevent the development of the bacilli; and secondly, to destroy the micro-organisms embedded in the pseudo-membranes. The author has studied the effect of a great number of medicaments on the cultures in blood serum. Of those, the following gave the best results: Solution of sublimate 1-10,000 destroyed the bacilli; by 1-1,000 the cultures were destroyed. Solutions of cyanide of mercury destroyed the bacilli in solution of 1-10,000: cultures 1-1,000. Nitrate of silver had effect in solutions of 1-1,000. Permanganate of potash 1-100, and chlorate of potash 1-20 were without effect. Iodine trichloride was effective in solution of 1-2,000. Absolute alcohol and ether destroyed the bacilli, but not the cultures. Chloroform water was very effective. Carbolic acid destroyed the bacilli in solutions of three to four per cent.; the cultures in five per cent. Salicylic acid was of less effect. Also many ethereal oils were tried and found more or less effective. The author concludes: For prophylactic use gargling with solution of sublimate 1-10,000, or aqua chloroformi. The steam of orange, citron, eucalyptus oil, &c., may be applied by means of Feldbausch's inhalers. For treatment of the disease, gargling with sublimate 1-1,000; carbolic acid 3-100. Turpentine is also recommended.

References: *Berl. Klin. Woch.*, Nos. 39 and 40; *Pract.*. April, 1891; *Deutsche Med. Woch.*, Nov. 10th, 1891; *Jour. of Laryng.*, 1891.

